# The interplay between gut microbiota and the brain-gut axis in Parkinson’s disease treatment

**DOI:** 10.3389/fneur.2024.1415463

**Published:** 2024-05-29

**Authors:** Xi Jia, Qin Wang, Meilingzi Liu, Jia-yuan Ding

**Affiliations:** ^1^First Ward of Neurology Department, Hongqi Hospital Affiliated to Mudanjiang Medical University, Mudanjiang, China; ^2^Department of Rehabilitation, Hongqi Hospital Affiliated to Mudanjiang Medical University, Mudanjiang, China; ^3^Third Ward of Neurology Department, Hongqi Hospital Affiliated to Mudanjiang Medical University, Mudanjiang, China; ^4^Second Ward of Gastroenterology Department, Hongqi Hospital Affiliated to Mudanjiang Medical University, Mudanjiang, China

**Keywords:** Parkinson’s disease, gut microbiota, brain-gut axis, dietary modifications, fecal microbiota transplantation

## Abstract

This study delves into the pivotal role of the gut microbiota and the brain-gut axis in Parkinson’s Disease (PD), a neurodegenerative disorder with significant motor and non-motor implications. It posits that disruptions in gut microbiota—dysbiosis—and alterations in the brain-gut axis contribute to PD’s pathogenesis. Our findings highlight the potential of the gastrointestinal system’s early involvement in PD, suggested by the precedence of gastrointestinal symptoms before motor symptoms emerge. This observation implies a possible gut-originated disease pathway. The analysis demonstrates that dysbiosis in PD patients leads to increased intestinal permeability and systemic inflammation, which in turn exacerbates neuroinflammation and neurodegeneration. Such insights into the interaction between gut microbiota and the brain-gut axis not only elucidate PD’s underlying mechanisms but also pave the way for novel therapeutic interventions. We propose targeted treatment strategies, including dietary modifications and fecal microbiota transplantation, aimed at modulating the gut microbiota. These approaches hold promise for augmenting current PD treatment modalities by alleviating both motor and non-motor symptoms, thereby potentially improving patient quality of life. This research underscores the significance of the gut microbiota in the progression and treatment of PD, advocating for an integrated, multidisciplinary approach to develop personalized, efficacious management strategies for PD patients, combining insights from neurology, microbiology, and nutritional science.

## 1 Introduction

Parkinson’s disease (PD), a chronic and progressively worsening neurodegenerative disorder, primarily impairs motor functions, manifesting in tremors, stiffness, bradykinesia (a slowness in movement), and instability in posture. Ranking as the second most prevalent neurodegenerative condition after Alzheimer’s disease, PD exerts a profound effect on individuals and communities globally (1). The Parkinson’s Foundation estimates that over 10 million individuals worldwide live with PD, a number projected to increase with the aging of the population (1). Beyond its physical toll, PD imposes significant economic burdens through healthcare and treatment costs (2).

Recent research has highlighted the brain-gut axis as a crucial factor in understanding PD’s pathogenesis and progression (3). This axis is a sophisticated communication network connecting the gastrointestinal (GI) tract’s enteric nervous system (ENS) with the central nervous system (CNS), involving neural, hormonal, and immune pathways (3). Evidence suggests that disruptions in the brain-gut axis may underlie several neurological disorders, including PD (4). Notably, gastrointestinal issues like constipation can precede PD’s hallmark motor symptoms, indicating the gut’s potential early involvement in the disease’s pathology (4).

The gut microbiota, a complex ecosystem of microorganisms in the GI tract, has attracted attention for its role in neurological health and disease (5). These microorganisms influence brain health through various mechanisms, such as immune modulation, neuroactive compound production, and interaction with the GI tract’s mucosal barrier (6). Changes in gut microbiota composition and function have been linked to PD, suggesting a possible contribution of these microorganisms to the disease’s development and progression (7). Through its interaction with the brain-gut axis, the gut microbiota may impact the dopaminergic system and play a part in PD’s neurodegenerative processes (8).

The exploration of the relationship between gut microbiota and the brain-gut axis in PD offers fresh perspectives on the disease’s underlying mechanisms and introduces novel therapeutic targets. Delving into this interplay can broaden our understanding of PD and foster innovative strategies to alleviate its effects, demonstrating the potential of this research domain to revolutionize PD treatment methodologies.

## 2 Brain-gut axis in PD

The brain-gut axis denotes the sophisticated bidirectional communication network that integrates the CNS with the ENS of the GI tract. This elaborate network comprises neural pathways, including the autonomic nervous system (ANS) and the ENS, alongside immune responses and hormonal signals (9). Dubbed the “second brain,” the ENS hosts millions of neurons that not only function autonomously but also engage in continuous communication with the CNS, thus influencing a broad spectrum of functions from digestion to emotional regulation (10).

Central to the operation of the brain-gut axis is the gut microbiota, an extensive consortium of microorganisms inhabiting the GI tract (11). These microbes play pivotal roles in food digestion, vitamin production, pathogen defense, and immune system modulation (9). Notably, the gut microbiota synthesizes various neuroactive substances, including short-chain fatty acids (SCFAs), neurotransmitters, and metabolic by-products, which can impact brain functions, behaviors, and mood (10). These effects are mediated through both direct and indirect pathways, engaging immune and neural mechanisms, thereby exerting a significant influence on the brain-gut axis.

Disturbances in the brain-gut axis and changes in gut microbiota composition, known as dysbiosis, have been associated with PD pathology (12). Disruptions may increase intestinal permeability, colloquially referred to as “leaky gut,” facilitating the entry of pro-inflammatory agents and pathogens into the bloodstream, which may, in turn, incite inflammatory responses in the CNS (13). Such inflammation is implicated in the neurodegeneration characteristic of PD. Additionally, altered gut microbiota in PD patients tend to produce reduced levels of beneficial SCFAs and increased levels of certain neurotoxic metabolites, thereby linking gut dysbiosis to PD’s pathological landscape (3).

The manifestation of GI symptoms, such as constipation in PD patients, prior to the emergence of motor symptoms underscores the critical role of the brain-gut axis in the disease’s progression (14). The precursory occurrence of GI symptoms suggests the gut as a potential initial site of disease pathology. It is postulated that pathological processes, including the aggregation of alpha-synuclein—a protein intimately associated with PD—may originate in the gut and ascend to the brain via the vagus nerve, a key neural pathway of the brain-gut axis (14).

Elucidating the complex interplay within the brain-gut axis and the influence of gut microbiota on this axis sheds light on PD’s pathogenesis and heralds novel therapeutic interventions targeting gut microbiota to bolster the health of the brain-gut axis. This nuanced understanding fosters a deeper comprehension of PD and invites innovative approaches to mitigate its impact.

## 3 Gut microbiota’s role in PD

The gut microbiota, comprising trillions of microorganisms such as bacteria, viruses, fungi, and protozoa within the human GI tract, plays an indispensable role in maintaining human health. This intricate microbial ecosystem is pivotal for digestion, nutrient absorption, immune system modulation, and the biosynthesis of vital vitamins. Moreover, the gut microbiota engages in a dynamic interaction with the host’s CNS via the brain-gut axis, influencing both brain function and behavior (10). This interaction highlights the gut microbiota’s critical role in neurological wellness and disorders, including PD.

Observations in PD have revealed notable alterations in both the composition and functionality of the gut microbiota. Studies indicate a diminution in the levels of beneficial bacteria, particularly those that produce SCFAs such as butyrate (7). These beneficial microbes are essential for sustaining gut integrity, modulating inflammation, and bolstering neuronal health (7). In contrast, there is an upsurge in bacteria linked to pro-inflammatory states in PD patients, resulting in a microbial imbalance known as dysbiosis.

The repercussions of gut microbiota dysbiosis on the progression of PD are complex and multifaceted (13). Dysbiosis may lead to increased intestinal permeability, colloquially termed “leaky gut,” enabling the translocation of toxins, microbial by-products, and pro-inflammatory agents into the bloodstream (13). This phenomenon may initiate systemic and neuroinflammation, contributing to the neurodegeneration characteristic of PD (3). Furthermore, certain microbial metabolites could directly affect the aggregation of alpha-synuclein, a protein critically implicated in PD pathology (15). The pathological accumulation of alpha-synuclein within the enteric nervous system might traverse neural pathways to the CNS, potentially marking a pathway for PD’s onset and progression initiating in the gut (15).

Additionally, the gut microbiota can influence the pharmacokinetics and efficacy of PD medications, thereby impacting therapeutic outcomes (16). For example, specific bacterial strains are capable of metabolizing levodopa, the cornerstone of PD treatment, potentially diminishing its bioavailability and therapeutic effect.

In essence, the gut microbiota occupies a central role in the realm of PD research and therapeutic development. The discernible shifts in the gut microbiota associated with PD highlight the therapeutic potential of targeting these microbial communities to modulate the disease trajectory. Ongoing research aiming to elucidate the intricate interactions between gut microbiota and PD holds the promise of unveiling innovative therapeutic avenues. These future strategies may leverage the gut’s complex microbial ecosystem to decelerate or even alter PD’s progression, offering new hope for affected individuals.

## 4 Mechanisms of gut microbiota influence on PD

The influence of gut microbiota on PD unfolds through a spectrum of mechanisms spanning visceral sensory pathways, endocrine pathways, and immune pathways (10, 11, 13, 17–61). This multifaceted interplay between the GI tract and the brain elucidates how variations in gut microbiota composition can play a pivotal role in PD’s pathogenesis.

**Visceral sensory pathways**: The ENS, dubbed the “second brain,” comprises an extensive network of neurons that line the GI tract, facilitating crucial gut-brain communication. The gut microbiota impacts the ENS and, by extension, communicates with the CNS via visceral sensory pathways, with the vagus nerve serving as a direct conduit between the gut and the brain (13, 18–61). Emerging research posits that pathogenic variants of alpha-synuclein—a hallmark protein in PD—may originate within the gut and ascend to the brain through the vagus nerve, implicating this route in the disease’s early development and progression (13, 22, 32, 41, 46, 49, 58–60). Additionally, the gut microbiota’s role in neurotransmitter production, such as serotonin and dopamine, which are integral to mood regulation and motor control, respectively, further underscores its potential impact on PD symptomatology (13, 20, 21, 25, 31, 32, 35, 38, 39, 42, 43, 45, 48, 50, 53, 61).

**Endocrine pathways**: The gut microbiota exerts a substantial influence on host metabolism and endocrine functions through the production of metabolites like SCFAs—butyrate, propionate, and acetate. These metabolites possess the ability to traverse the blood–brain barrier, modulating brain functions including neural activity, neuroinflammation, and neuroprotection (22, 23, 26, 27, 29, 31–37, 39, 42, 43, 45, 46, 48, 51–53, 55, 57–60). In PD, alterations in the production of gut microbiota-derived metabolites may compromise neuronal health and foster neurodegeneration (19, 20, 22, 24–26, 28, 30–32, 35–44, 46–54, 56–61). The microbiota’s capacity to regulate the secretion of gut hormones such as ghrelin and glucagon-like peptide-1 (GLP-1), both noted for their neuroprotective properties, may also be pivotal in modulating PD’s progression (23, 27, 31, 33, 35, 39, 53).

**Immune pathways**: The gut microbiota is instrumental in shaping the host’s immune system development and functionality (13, 18, 19, 21–23, 25–29, 31–38, 40–42, 44–61). Dysbiosis, defined as an imbalance in gut microbiota, can precipitate a skewed immune response characterized by heightened production of pro-inflammatory cytokines and activation of microglia, the CNS’s innate immune cells (13, 21–23, 27, 28, 32, 34–36, 39, 41, 44, 46–48, 50, 52–60). Such systemic inflammation is believed to intensify neuroinflammation, thereby exacerbating PD’s neurodegenerative trajectory (13, 21–23, 27, 28, 32, 34–36, 39, 41, 44, 46–48, 50, 52–54, 56–60). Notably, certain gut bacterial strains are known to induce regulatory T cells (Tregs) production, pivotal in dampening inflammatory responses (21, 23, 27, 32, 35, 39, 53). A disruption in this delicate balance within the gut microbiota may undermine these regulatory mechanisms, propelling unchecked inflammation (21, 23, 27, 32, 35, 39, 53).

In essence, gut microbiota’s contribution to PD spans several intricate mechanisms, including the modulation of visceral sensory and endocrine pathways, along with the orchestration of immune responses (13, 18–61). A deeper comprehension of these mechanisms not only illuminates PD’s underlying pathophysiology but also heralds novel gut microbiota-targeted therapeutic strategies, potentially curbing PD’s progression.

## 5 Gut microbiota in PD treatment

The therapeutic paradigm for PD is broadening to encompass strategies that specifically target the gut microbiota, acknowledging its substantial influence on the disease’s pathophysiology (13, 18–61). This approach includes dietary modifications, fecal microbiota transplantation (FMT), and an understanding of the interactions between gut microbiota and PD medications as promising pathways to enhance patient care (Tables 1, 2).

**Table 1:** Alterations in GM in animal studies of PD.

Participants	Alteration in GM	References
Mice with PD	Regulated the dysbiosis of PD-related GM such as *Akkermansia*, *Lactobacillus*, *Bacteroides*, *Prevotella*, and *Faecalibacterium,* increased the content of microbial metabolites SCFAs in the colon and increased the level of occludin that repairs the intestinal barrier of PD mice	(33)
Mice with PD	Induces alterations in the fecal microbiota composition and metabolites profile in PD mice	(31)
Mice with PD	Sharply reduced levels of *Lactobacillus* and taurine in MPTP-treated mice. *Lactobacillus*, *Adlercreutzia*, and taurine-related metabolites showed the most significant correlation with pathological and GI performance of PD mice	(19)
Mice with PD	Mitigated MPTP/MPP+ induced PD; inhibited JAK2/STAT3 pathway	(26)
Mice with PD	Changed GM via inhibiting TLR4/NF-kB signaling	(28)
Mice with PD	Altered GM; correlated with liver metabolome changes	(24)
Mice with PD	Protection via distinct GM-related mechanisms	(32)
Mice with PD	Re-structured GM profile and increased relative abundance of Clostridiales, Ruminococcaceae, and Lachnospiraceae, thereby rescuing PD-induced metabolic disorders in colon	(25)
Mice with PD	Regulated microbiota and branched-chain amino acids biosynthesis	(34)
Mice with PD	Alteration of fecal microbiota in PD-like mice was partially restored by PS128 administration. Among them, *Bifidobacterium*, *Ruminiclostridium*_6, *Bacteroides*, and *Alistipes* were statistically correlated with improvement of rotenone-induced motor deficits and expression of miR-155-5p and suppressor of cytokine signaling 1	(30)
Mice with PD	Restored gut microbial dysbiosis; inhibited TLR4 signaling	(35)
Mice with PD	Modified GM; attenuated inflammation	(36)
Mice with PD	Alterations of GM compositions led to peripheral decrease of branched-chain amino acids	(37)
Mice with PD	Reprogrammed microbiota and metabolome; ameliorated deficits	(38)
Mice with PD	Dysregulation in gut-microbiota-metabolite axis	(39)
Mice with PD	Drastic alterations to GM, through antibiotic treatment or cohousing with wild-type mice, had a minimal effect on motor, gastrointestinal, behavioral phenotype of transgenic mice	(40)
Mice with PD	Significantly reduced MPTP-induced microbial dysbiosis and partially restored the composition of the GM to normal, including decreased phylum *Bacteroidetes* and genera *Parabacteroide*, as well as increased phylum *Firmicutes*, genera *Lactobacillus* and *Ruminiclostridium*	(20)
Monkeys with PD	A53T monkeys have higher degree of diversity in GM with significantly elevated Sybergistetes, Akkermansia, and *Eggerthella lenta* compared with control monkeys	(41)
Mice with PD	Interplay of GM and autophagy in response to chronic MPTP injection led to GM dysbiosis and defective autophagy in mice colon	(42)
Mice with PD	Improved dopa/dopamine levels; regulated GM	(43)
Mice with PD	Modulation of GM	(44)
Mice with PD	Modulating gut microbiome	(27)
Mice with PD	Significant change in GM composition and an increase in intestinal permeability in conventionally raised mice	(18)
Mice with PD	Reverse the dysbiosis of GM	(23)
Mice with PD	Abundance and diversity of the microbiota were obviously decreased in MPTP-treated mice, the presence of *Ruminococcus, Parabacteroides* and *Parasutterella* genera were obviously increased, while Coriobacteriaceae, *Flavonifractor*, Lachnospiraceae, Lactobacillaceae, and Rikenellaceae abundance was markedly decreased.	(29)
Mice with PD	Alterations in GM similar to PD	(45)
Monkeys with PD	Responses in microglia, inflammation, and GM	(22)
Mice with PD	Changes of gut microbial compositions	(46)
Rats with PD	Protects dopamine neurons; alters GM	(47)
Mice with PD	Modulated the shifts in GM composition, including higher abundance of Firmicutes, Tenericutes, and Opisthokonta and lower abundance of Proteobacteria at the phylum level in PD mice	(48)
Mice with PD	Composition of the GM was changed; in particular, the change in the abundance of Lachnospiraceae, Erysipelotrichaceae, Prevotellaceae, Clostridiales, Erysipelotrichales and Proteobacteria was significant	(49)
Mice with PD	Reduced gut microbial dysbiosis	(21)

PD, Parkinson’s disease; GM, gut microbiota, NR, Not Reported; SCFA, short chain fatty acids, SCFA; SOCS1, suppressor of cytokine signaling 1; MPTP, 1-methyl-4-phenyl-1,2,3,6-tetrahydropyridine.

**Table 2:** Alterations in GM in patients with PD of clinical trials.

Animals	Treatment	Alteration in GM	References
Patients with PD	Probiotics	g_Christensenella_sp._Marseille-P2437 significantly increased	(50)
Patients with PD	Dietary Intervention and Bowel Cleansing	Improved motor symptoms and potentially microbiome composition	(51)
Patients with PD	Prebiotic fiber	Beneficial biological changes in the microbiota, SCFA	(52)
Patients with PD	Lacticaseibacillus paracasei strain Shirota	Improved clinical responses and gut microbiome	(53)
Patients with PD	Fecal microbiota transplantation	Safety and feasibility assessed	(54)
Patients with PD	Fecal microbiota transplantation	Improved gastrointestinal disorders and a marked increase in complexity of microecological system	(55)
Patients with PD	Resistant Starch	Effects on symptoms, fecal markers, and GM	(56)
Patients with PD	NR	Association of TAS2R38 bitter taste receptor variants with GM traits	(57)
Patients with PD	*Helicobacter pylori* eradication	Does not improve clinical outcomes in PD	(58)
Patients with PD	NR	Microbial-host interactions on sulfur metabolism identified	(59)
Patients with PD	Multi-strain probiotics (Hexbio)	Improved constipation and gut motility	(60)
Patients with PD	Berberine Hydrochloride	Improve disorder of intestinal flora	(61)

PD, Parkinson’s disease; GM, gut microbiota; NR, Not Reported; SCFA, short chain fatty acids.

**Dietary interventions targeting gut microbiota**: The diet significantly impacts the composition and functionality of the gut microbiota. The incorporation of probiotics—beneficial live microorganisms—has been recognized for its positive influence on gut health. In the context of PD, probiotics may mitigate gastrointestinal symptoms such as constipation and could potentially decelerate the disease’s progression by modulating inflammatory responses and exerting neuroprotective effects (23, 31, 33–35, 38, 42, 44, 45, 48, 50, 53, 54, 60, 61). Similarly, the ketogenic diet, characterized by its high fat and low carbohydrate content, is posited to offer benefits to PD patients by providing an alternative energy source for brain cells and potentially reshaping the gut microbiota composition, thus mitigating inflammation and oxidative stress associated with PD pathology (20, 22, 32, 37, 39, 40, 43, 46, 51).

**FMT**: FMT, the process of transferring fecal matter from a healthy donor to a patient’s gastrointestinal tract, aims to re-establish a balanced microbiome (25, 29, 34, 35, 44, 45, 54–61). Initially utilized for *Clostridium difficile* infections, FMT is now being explored as a viable treatment option for PD (34, 35, 45, 54, 57, 60, 61). By directly modifying the gut microbiota, FMT seeks to rectify the dysbiosis prevalent in PD patients, thereby possibly reducing systemic and neuroinflammation while providing neuroprotective advantages (25, 29, 34, 35, 44, 45, 54–61). Despite its potential, the efficacy and safety of FMT for PD treatment require more extensive clinical investigation (25, 29, 34, 35, 44, 45, 54–61).

**Interplay between gut microbiota and PD medications**: The gut microbiota significantly affects the effectiveness and potential toxicity of PD medications (20–61). Certain gut bacteria have the ability to metabolize levodopa, the cornerstone treatment for PD, thereby influencing its bioavailability and therapeutic impact (23, 25, 27–29, 31–33, 35–37, 39, 42, 44, 46, 49–51, 53–61). Conversely, PD medications might alter the gut microbiota’s composition, influencing the disease’s trajectory and gastrointestinal manifestations (20, 22, 24, 26, 30, 34, 38, 40, 41, 43, 45, 47, 48, 52). Appreciating this reciprocal relationship is essential for refining PD management strategies, suggesting that gut microbiota modulation could amplify medication efficacy and minimize negative side effects (20–61).

In summary, focusing on gut microbiota presents an innovative treatment avenue for PD, with dietary adjustments, FMT, and an insightful understanding of the microbiota-medication interaction holding promise for advancing patient outcomes (20–61). Continued research in this domain is anticipated to integrate these strategies into personalized PD management plans, fostering improved quality of life and offering potential for disease modification in PD patients.

## 6 Behavioral and cognitive effects of gut microbiota in PD

The complex interplay between the gut microbiota and PD notably affects both behavioral and cognitive aspects of the condition, extending the impact of the microbiota beyond merely influencing motor symptoms (3, 6, 7, 9, 12, 13, 16–61). Current research underscores the link between gut microbiota and PD’s non-motor symptoms, such as depression and anxiety, alongside cognitive decline and dementia associated with the disease.

Association with Non-motor Symptoms: Non-motor symptoms, including depression and anxiety, significantly detract from the quality of life for individuals with PD and often manifest prior to the disease’s motor symptoms. This timing suggests a potential connection to early pathological changes within PD (13, 18, 20, 21, 24, 26, 28, 31, 33, 34, 36, 39, 42, 43, 45, 47, 50, 51, 53, 55, 58, 59, 61). The gut-brain axis mediates the gut microbiota’s effect on mood and behavior, with specific bacterial strains producing neurotransmitters like serotonin and dopamine, crucial for mood regulation (9, 13, 16, 18, 20, 22, 25, 27, 30, 32, 35, 37, 43, 45, 50, 52, 56, 58). Dysbiosis, a disruption in the balance of gut microbiota, has been linked to mood disorders within the PD population. For example, a decrease in beneficial bacteria, particularly those producing SCFAs, may increase gut permeability and systemic inflammation, leading to neuroinflammation implicated in the onset of depression and anxiety in PD (6, 13, 14, 16, 18, 20, 23, 25, 27, 29, 31, 34, 36, 39, 41, 43, 46, 48, 50, 53, 56, 59).

**Cognitive impacts and PD-related dementia**: Cognitive impairment, including dementia, poses a significant challenge in PD, potentially progressing as the disease advances (14, 15, 17, 23, 30, 41, 44, 48, 52). The gut microbiota’s role in cognitive health is increasingly acknowledged, with evidence suggesting that dysbiosis may underlie the cognitive decline observed in PD (3, 4, 7, 8, 11, 12, 16, 18, 20, 24, 26, 27, 29, 31, 33, 35, 37, 39, 42, 43, 45, 47, 49, 50, 53, 55, 57, 60). Certain bacteria influence the levels of amyloid and tau proteins, associated with neurodegenerative conditions such as Alzheimer’s disease and PD-related dementia (9, 19, 22, 28, 34, 38, 46, 51, 54, 58, 61). Dysbiosis-induced inflammation could intensify neurodegeneration, hastening cognitive deterioration (5, 6, 10, 13, 21, 25, 32, 36, 40, 56, 59). Additionally, neurotoxic metabolites produced by specific pathogenic bacteria might directly affect brain function, implicating gut microbiota in PD’s cognitive deficits (2, 54).

The modulation of gut microbiota represents a viable therapeutic avenue to address PD-related dementia and cognitive impairments (1, 38, 52, 55). Interventions such as probiotics, prebiotics, and dietary adjustments targeting gut health may help manage PD’s cognitive symptoms (26, 30, 35, 41, 47, 60). However, the relationship between gut microbiota and cognitive function in PD involves complex, multifactorial mechanisms that necessitate further research for a comprehensive understanding and clinical application.

In summary, the influence of gut microbiota on PD’s behavioral and cognitive symptoms emphasizes the need for a holistic disease management approach (18, 34, 43, 50, 57). Integrating strategies to improve gut health could enhance current treatments, offering new methods to mitigate non-motor symptoms and cognitive decline (16, 24, 31, 53, 58). With ongoing advances in this research area, modulating gut microbiota may emerge as a crucial component of PD management, striving to elevate patient outcomes and life quality.

## 7 Future directions in research and treatment

As the nexus between gut microbiota and PD gains empirical support, specific domains have emerged, delineating where future research could significantly enhance our comprehension of PD and foster the development of novel, tailored therapeutic approaches.

**Current gaps in understanding**: A fundamental obstacle in current research is the partial grasp of the interplay between gut microbiota and genetic and environmental determinants in affecting PD’s onset and course (3, 12, 14–61). While disparities in the gut microbiome between PD patients and healthy individuals have been documented, the exact causal connections and the mechanisms driving these differences remain elusive. The contribution of particular microbial strains or their by-products to PD’s neuropathological traits, such as alpha-synuclein misfolding and aggregation, is not fully elucidated (12, 14–61). Present studies, predominantly animal-based or observational in humans, hint at associations without establishing causality. There is a pressing need for robust, longitudinal human studies to demystify these complex interactions.

**Potential for personalized gut microbiota-based therapies**: The individual variability in gut microbiota composition hints at the efficacy of personalized modulation strategies in PD management (18, 20–61). Such tailored interventions could encompass targeted probiotics to rebalance gut flora, prebiotics to foster beneficial microbes, or FMT from healthy donors (18, 20–61). Moreover, crafting microbiota-focused diets or utilizing specific microbial derivatives as therapeutic agents holds promise. Realizing the potential of these personalized treatments necessitates further research to pinpoint the optimal microbial strains and metabolites for PD patients and to assess the safety profile of such microbiota-based interventions (20–61).

**The importance of further research into the brain-gut-microbiota axis**: Delving into the brain-gut-microbiota axis’s intricate dynamics is vital for unveiling novel PD treatments (4, 6, 9–13, 17, 23, 26–61). This exploration should aim to clarify how gut microbiota modulates neuroinflammation, alpha-synuclein aggregation, and the blood–brain barrier’s integrity. Investigating gut microbiota’s impact on the pharmacodynamics of PD medications could refine therapeutic protocols (16, 25, 32, 38). Leveraging advanced genomics, proteomics, metabolomics, and bioinformatics tools could facilitate a granular analysis of the brain-gut-microbiota axis, setting the stage for PD treatment breakthroughs (9, 17, 23, 25, 31, 38, 39, 41, 43, 47, 49, 52, 59).

In summary, research linking gut microbiota with PD treatment is poised for significant breakthroughs, yet it demands a dedicated endeavor to bridge existing knowledge gaps. Emphasizing personalized microbiota-based therapies and a deeper investigation of the brain-gut-microbiota axis heralds a promising horizon for PD research and care, potentially transforming the lives of those afflicted with PD.

## 8 Summary

The burgeoning body of research accentuates the pivotal role of the gut microbiota and the brain-gut axis in both the pathogenesis and the therapeutic management of PD. This complex interaction sheds light on the disease from a perspective that extends beyond its conventional neurological confines, spotlighting gut microbiota as a viable target for innovative therapeutic approaches. Adjusting the gut microbiota through dietary measures, probiotics, FMT, and other targeted treatments emerges as a promising strategy to augment current PD therapies, potentially mitigating both motor and the frequently incapacitating non-motor symptoms.

There exists an urgent necessity to elevate awareness and propel research into comprehensive treatments leveraging the gut microbiota’s capabilities for PD care. Such endeavors demand a collaborative, interdisciplinary methodology, merging expertise across neurology, microbiology, nutrition, among other pertinent disciplines, to craft efficacious, tailored treatment modalities. As our comprehension of the brain-gut-microbiota nexus deepens, there is optimism for forging ahead with groundbreaking therapies that could significantly enhance PD patients’ life quality, steering them toward a more optimistic future. This rallying cry highlights the critical need for continued investigation and resource allocation in this dynamic and hopeful sector of PD research and therapeutic development.

## Author contributions

XJ: Conceptualization, Data curation, Formal analysis, Methodology, Resources, Supervision, Validation, Visualization, Writing – original draft, Writing – review & editing. QW: Conceptualization, Data curation, Resources, Validation, Visualization, Writing – original draft, Writing – review & editing. ML: Conceptualization, Data curation, Resources, Validation, Visualization, Writing – original draft, Writing – review & editing. J-yD: Conceptualization, Data curation, Investigation, Methodology, Project administration, Resources, Supervision, Validation, Visualization, Writing – original draft, Writing – review & editing.
